# The Identification of Bee Comb Cell Contents Using Semiconductor Gas Sensors

**DOI:** 10.3390/s23249811

**Published:** 2023-12-14

**Authors:** Beata Bąk, Jakub Wilk, Piotr Artiemjew, Maciej Siuda, Jerzy Wilde

**Affiliations:** 1 Department of Poultry Science and Apiculture, Faculty of Animal Bioengineering, University of Warmia and Mazury in Olsztyn, Sloneczna 48, 10-957 Olsztyn, Poland; beata.bak@uwm.edu.pl (B.B.); teofil.wilk@uwm.edu.pl (J.W.); maciej.siuda@uwm.edu.pl (M.S.); 2 Faculty of Mathematics and Computer Science, University of Warmia and Mazury in Olsztyn, 10-719 Olsztyn, Poland

**Keywords:** bee cells, bee honey, bee brood, gas sensors, classification

## Abstract

Beekeeping is an extremely difficult field of agriculture. It requires efficient management of the bee nest so that the bee colony can develop efficiently and produce as much honey and other bee products as possible. The beekeeper, therefore, must constantly monitor the contents of the bee comb. At the University of Warmia and Mazury in Olsztyn, research is being carried out to develop methods for efficient management of the apiary. One of our research goals was to test whether a gas detector (MCA-8) based on six semiconductor sensors—TGS823, TGS826, TGS832, TGS2600, TGS2602, and TGS2603 from the company FIGARO—is able to recognize the contents of bee comb cells. For this purpose, polystyrene and wooden test chambers were created, in which fragments of bee comb with different contents were placed. Gas samples were analyzed from an empty comb, a comb with sealed brood, a comb with open brood, a comb with carbohydrate food in the form of sugar syrup, and a comb with bee bread. In addition, a sample of gas from an empty chamber was tested. The results in two variants were analyzed: (1) Variant 1, the value of 270 s of sensor readings from the sample measurement (exposure phase), and (2) Variant 2, the value of 270 s of sensor readings from the sample measurement (measurement phase) with baseline correction by subtracting the last 600 s of surrounding air measurements (flushing phase). A five-time cross-validation 2 (5xCV2) test and the Monte Carlo cross-validation 25 (trained and tested 25 times) were performed. Fourteen classifiers were tested. The naive Bayes classifier (NB) proved to be the most effective method for distinguishing individual classes from others. The MCA-8 device brilliantly differentiates an empty comb from a comb with contents. It differentiates better between an empty comb and a comb with brood, with results of more than 83%. Lower class accuracy was obtained when distinguishing an empty comb from a comb with food and a comb with bee bread, with results of less than 73%. The matrix of six TGS sensors in the device shows promising versatility in distinguishing between various types of brood and food found in bee comb cells. This capability, though still developing, positions the MCA-8 device as a potentially invaluable tool for enhancing the efficiency and effectiveness of beekeepers in the future.

## 1. Introduction

The nest of a bee colony is extremely aromatic. The smell emanating from the hive is quite pleasant to the human nose and is often associated with the scent of honey. Beekeepers tending to bee colonies greatly appreciate the smell of the hive’s air, as it has a soothing effect on the nervous system, relieves stress, and can be somewhat addictive. Because of this, many beekeepers continue to operate their apiaries into old age, and only the loss of physical fitness causes them to retire from beekeeping. Hive air has been used successfully in the treatment of respiratory diseases [1].

The scent emitted from a bee colony is created by a mixture of various volatile organic compounds released by the components of the nest. Its primary structure is the bee combs made of beeswax produced by the wax glands of worker bees. In the case of freshly built combs, it is the smell of this substance that predominates [2]. About 20 volatile substances, mainly belonging to alkanes, are responsible for this aroma. The most dominant compounds are 2,4-dimethyl-heptane, acetal, nonanal, and 4-methyl-octane [3].

Bee combs are made up of hexagonal cells in which bees store food in the form of honey and bee bread. The honeys produced by bees and stored in the cells of combs are particularly rich in various essential oils, phenols, alcohols, carboxylic acids, aldehydes, ketones, hydrocarbons, and polyphenols. About 80 aromatic substances have been identified in honey [4]. Their presence and ratio determine the organoleptic properties of honey. The predominant aromatic compounds in honey are benzaldehyde, furfural, and linalool [3]. Twenty-nine aromatic substances have been determined in bee bread, of which 24 are sulfides (dimethylsulfide, dimethyldisulfide, and dimethyltrisulfide).

Also, the queen lays eggs into the bee cells, which give rise to larvae and then pupae. All developmental forms of bees are called bee brood. The first 9 days of development of worker bees take place in open cells, in which case we speak of open brood, and the next 12 days are in cells closed with wax, in which case we speak of sealed brood. Bee brood is one of the components of a bee colony. Both open bee brood and sealed brood secrete a complex of substances called brood pheromones [5]. The composition of this signaling complex has been shown to be a mixture of 10 methyl or ethyl esters of higher C16 and C18 fatty acids (palmitic, stearic, oleic, linolenic, and linoleic) [6]. They are formed in the salivary glands of larvae and persist on the surface of the larvae’s cuticles [7]. These substances are non-volatile and often perishable, as they are quickly oxidized. The brood pheromone can have different compositions and combinations of esters, and this depends on what function it performs [8,9,10]. Additional odor components of open brood are larvae feces as well as royal jelly [11,12], in which 1–3 day-old larvae are immersed [7,13]. In the case of sealed brood, an additional odor element will be the wax capping.

Bee cells are covered by worker bees with a thin layer of propolis before the queen bee lays her eggs into them. The more generations of bee brood are raised in a comb, the more layers of propolis are found in its cells, which affects the thickness of the cell walls as well as the aroma of the comb. Propolis, otherwise known as bee putty, is the chemically richest bee product. It contains about 350 substances, but its composition is never identical. Most of its substances are resins (50–80%), and essential oils account for 4–15% of the products [14,15,16,17,18]. Thus, older slices are enriched with aroma components of the mentioned elements [18,19,20,21], which can significantly differentiate the odors of freshly built patches from old patches.

Apiary management requires constant inspections of bee colonies to determine their status and condition. Sometimes, weather conditions do not allow the opening of a bee nest, or the beekeeper lacks the time and opportunity to get to the apiary. This could be helped by semiconductor gas sensors that could conduct analysis of volatile odorous substances released from the nest and thus inform the beekeeper of the situation in the apiary, therefore streamlining work and reducing the cost of running the apiary. Air quality measurements using semiconductor gas sensors are already being carried out in many industries [22,23,24]. Excellent results were obtained in the detection of bee and brood diseases and the status of worker bees [25,26,27,28]. A particular application is FIGARO’s TGS series of sensors. The measuring element in these sensors is an electrode made of tin dioxide (SnO${}_{2}$). The working temperature of SnO${}_{2}$-based semiconductor sensors is 300–450 °C [29]. As a result of the contact of this semiconductor with volatile organic substances, a change in the concentration of current carriers occurs which, as a result, causes disturbances in the conductivity of the semiconductor expressed by a numerical value in units of volts. Several to dozens of sensors are used for qualitative measurement of air [30]. Each individual sensor responds differently to the presented gas sample, resulting in a configuration of signals of the entire matrix, which gives us a picture of the smell of a particular gas sample.

The purpose of our research was to find out whether semiconductor gas sensors are able to distinguish the odors of individual components of a bee nest, and thus we wanted to find out whether a device based on a matrix of these sensors is able to recognize the contents of the bee cells of a comb.

## 2. Materials and Methods

### 2.1. Laboratory Stand: Construction

**Figure 1:**
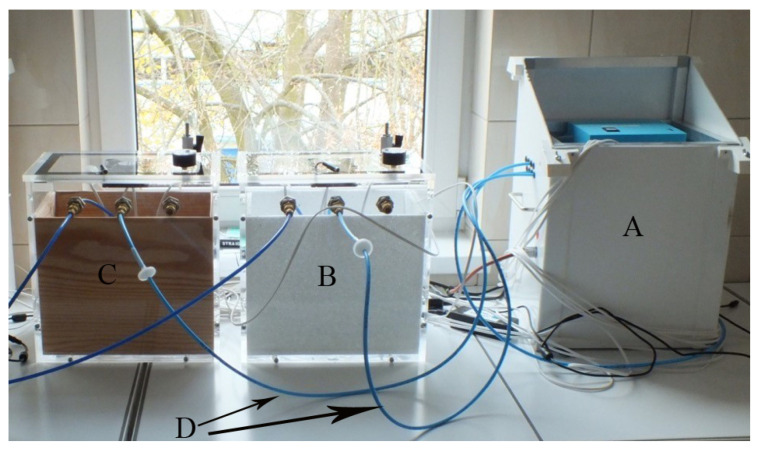
The laboratory stand: (**A**) gas detector (MCA-8), (**B**) Styrofoam chamber, (**C**) wooden chamber, and (**D**) polystyrene tubes.

**Figure 2:**
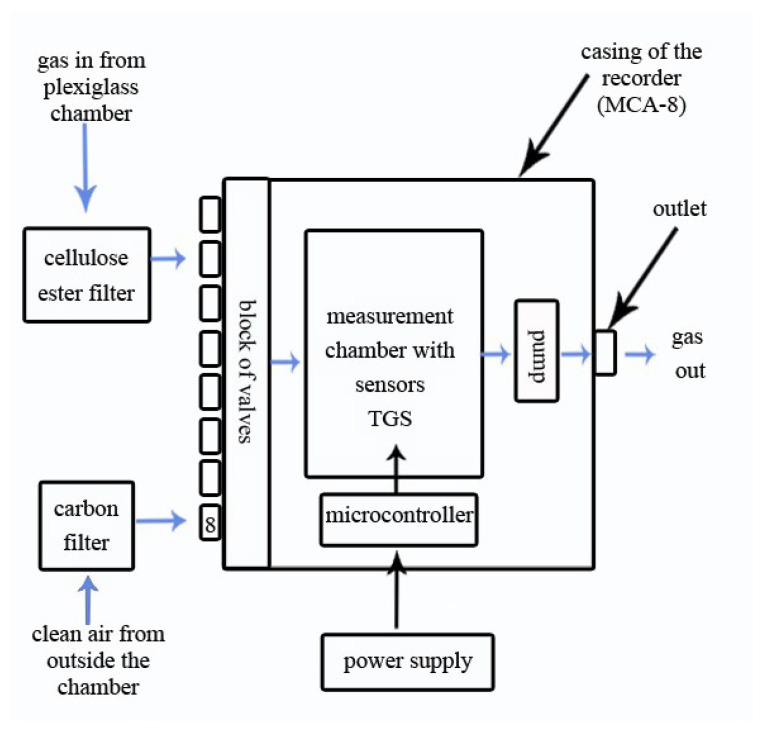
A gas sample flow diagram of detector MCA-8.

The experiment was carried out at the Bee Products Quality Monitoring and Safety Laboratory of Department of Poultry Science and Apiculture in the Faculty of Animal Bioengineering at the University of Warmia and Mazury in Poland. In order to carry out the tests, a special laboratory station was created which was constructed with two test chambers: a styrofoam one and a wooden one, as well as a measuring device connected to the chambers by polystyrene tubes (Figure 1). Styrofoam and wood were intended to mimic the environment of a bee colony, as hives are constructed from these materials. The measuring device was a gas detector (MCA-8) based on 6 semiconductor sensors: TGS823, TGS826, TGS832, TGS2600, TGS2602, and TGS2603 from the company FIGARO (Table 1). This device was developed in the Laboratory of Sensor Technique and Indoor Air Quality Studies at Wroclaw University of Science and Technology in Poland. The structure and operating diagram of the device are shown in Figure 2. The MCA-8 gas detector was equipped with an SD card reader, on which the data were recorded with a temporal resolution of 1 s and a GSM modem, which allowed data to be sent to a server (5 s resolution). This made it possible to analyze the data on the spot from the website without having to remove the SD card. Such a solution protects against data loss if the device is damaged. The data to another device could be transferred using an SD card. The device received its operating parameters via a configuration file placed on an SD card.

The MCA-8 device was designed so that the gas sensor heater’s temperature was stabilized individually for each sensor. For this purpose, the power applied to the sensor heater was controlled by the duty cycle of the pulse-width modulation signal (PWM). The range of this parameter was from 0% to 100%, and 100% was recommended by the producer. In addition, a sensor was installed to control the temperature of the aluminum block that housed the sensor chamber. An analog-to-digital conversion (ADC) resolution of 12 bits with oversampling to 16 bits in the successive approximation register (SAR) was used. This made it possible to obtain electronic circuits for measuring the sensor resistance and supervising heater operation according to sensor technical sheets. The MCA-8 device was equipped with temperature and humidity sensors. During testing in the laboratory, we tried to keep the temperature at a constant 22 °C. However, the results of the measurements conducted by the device show that the temperature fluctuated between 21.16 °C and 23.67 °C. The humidity was around 40% and ranged from 35.27% to 42.84%. The device was dedicated to continuous work and taking measurements in dynamic mode. A miniature diaphragm pump manufactured by NITTO KOHKI was used to force the gas flow. The maximum free gas flow rate for the pump was 5 L/min. During the measurements, the pump power was set to 50%.

Each day, before the measurements began, the device was heated up. For this purpose, it was operated for 1 h at 100% heater power without the pump on. Then, measurements were carried out throughout the day continuously to avoid overcooling of the sensors. Gas flow occurred on one of the seven working channels, and every two days, the channel was changed to a different one. Clean air from outside the chamber was drawn in through the eighth channel, which it was intended for, and passed through a carbon filter (Figure 2).

### 2.2. The Classes and Research Material

Six classes were created for comparison:

- Class 1: empty chamber;
- Class 3: comb with sealed bee brood;
- Class 4: comb with opened bee brood;
- Class 15: empty comb;
- Class 16: comb with carbohydrate bee food (sugar syrup);
- Class 17: comb with bee bread.

The fragments of combs with brood with an area of a minimum of 50 cm${}^{2}$ were taken from clinically healthy colonies. Prior to testing, they were cleaned and dried from the remains of damaged brood and then stored in an incubator (35 ${}^{\circ }$C). Each brood sample came from a different bee colony but from the same apiary. The fragments of empty combs and combs with food were of an area of a minimum of 50 cm${}^{2}$. In order to avoid botanical odors, the food stored in the cells of the patch was sugar syrup.

### 2.3. The Measuring Procedure

**Figure 3:**
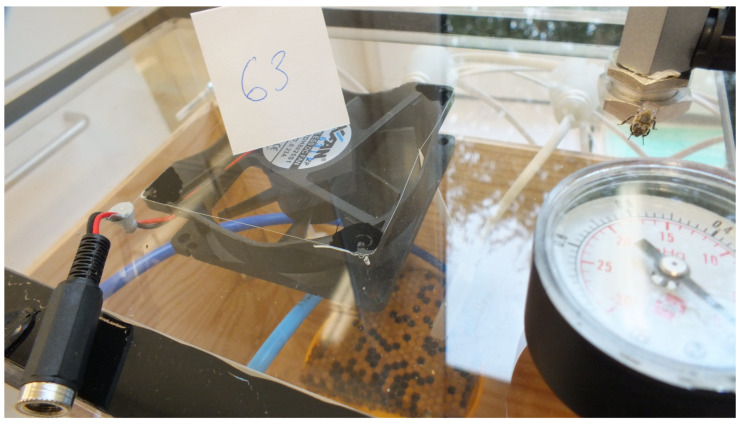
Sample of comb with sealed brood in wooden chamber during measurement.

For the measurement, the study sample was placed centrally in the test chamber so that the tip of the gas sampling tube was no more than 10 cm above the sample (Figure 3). The measurement lasted 20 min. The first 10 min was the measurement phase. In this stage, the gas was taken through the device’s inlet channel from the test chamber. The next 10 min was the sensor flushing phase. In this stage, the gas was taken through the device’s inlet in the eighth channel from outside the test chamber. A cellulose ester filter was placed in the way of gas taken from above the test sample, and a carbon filter was placed in the way of clean air taken from outside the test chamber (Figure 2).

### 2.4. Data Processing

**Figure 4:**
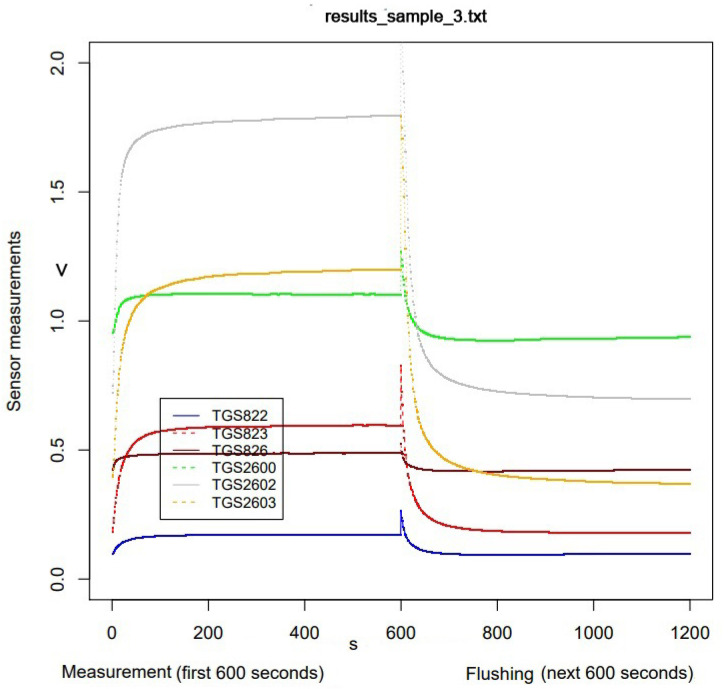
Example of visualization of a properly run measurement (open bee brood sample).

**Figure 5:**
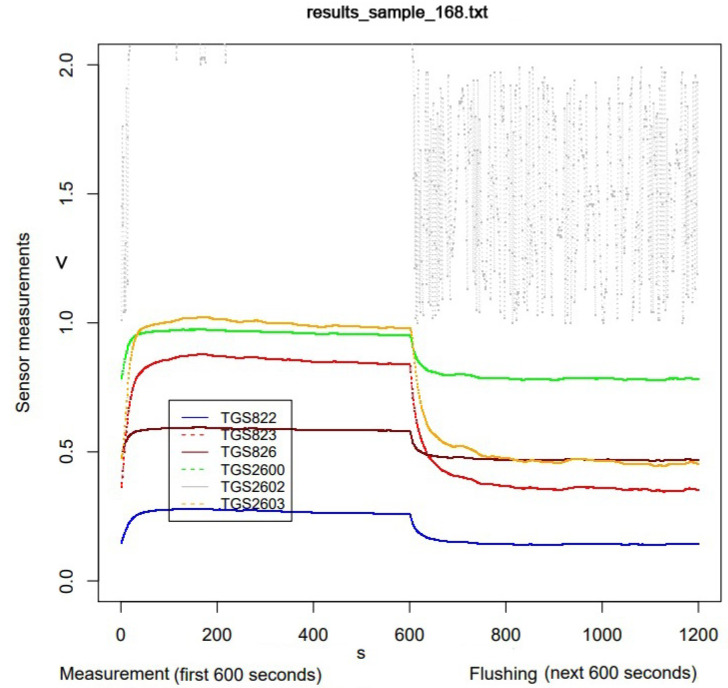
Example of visualization of a faulty run measurement (empty comb sample).

As part of the preselection of the data obtained, the sensor readings were visualized (Figure 4). It turned out that some of the charts looked incorrect (Figure 5), and these were rejected from further analysis.

As a result, only in class 16 could the measurement files of all samples be taken for analysis, and in the other classes, the number of samples analyzed was less than the number of samples measured correctly (Table 2).

## 3. Variant of Data Procesing and Analysis

The results in two variants were analyzed: (1) Variant 1, with a value of 270 s for sensor readings from the sample measurement (exposure phase), and (2) Variant 2, with a value of 270 s for sensor readings from the sample measurement (measurement phase) with baseline correction by subtracting the last 600 s of the surrounding air measurement (flushing phase).

A five-times cross validation 2 (5xCV2) test was performed. We performed additional tests in five-times Monte Carlo cross validation 25 (trained and tested 25 times) [31,32]. The TRN data partition coefficient was set to TST (0.7–0.3). Fourteen classifiers were tested:

- K nearest neighbor methods with the selected metrics;
- Naive Bayes classifier (NB) [33,34,35];
- SVM classifiers with appropriate kernels (linear, radial, polynomial, and sigmoid) [36];
- Classification based on logistic regression [37];
- Random forest method [38];
- Decision trees;
- Com3 [39], with a committee of selected classifiers.

Due to the fact that the samples were limited in availability, the findings are supported by radar plot visualizations to illustrate the ability to distinguish between classes. For this reason, classification was performed in a one vs. all model, where we confronted a different central class with the others each time or used a class pair strategy.

## 4. Results

**Figure 6:**
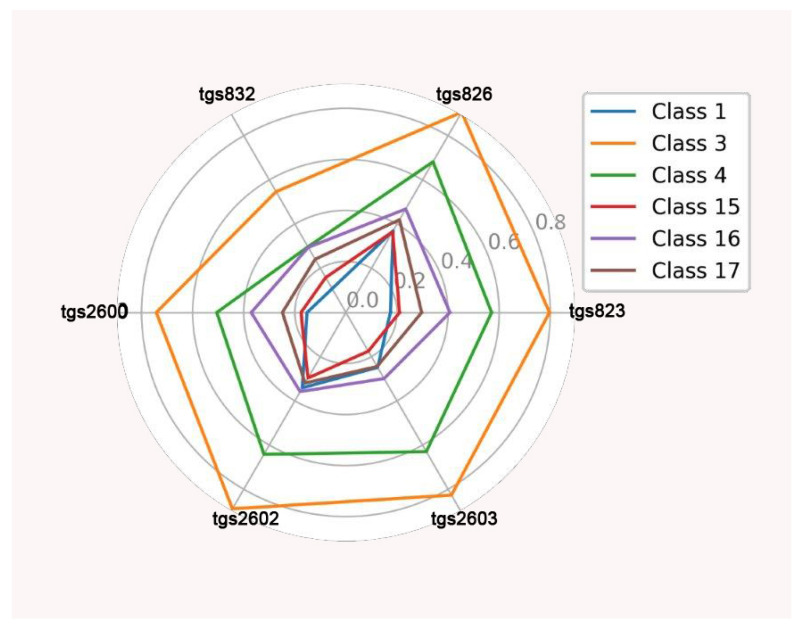
Average individual sensor readings for each class with baseline correction in the wooden chamber (V).

**Figure 7:**
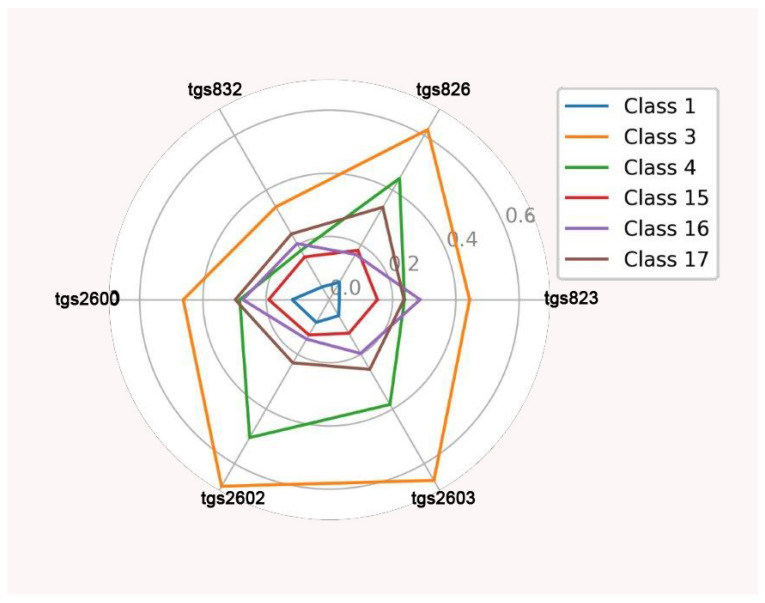
Average individual sensor readings for each class with baseline correction in the Styrofoam chamber (V).

The starting point for investigating classification possibilities is to visualize the average readings of the individual TGS sensors with the baseline correction. Visualization on radial graphs indicated a clear separability of classes. The highest average readings for individual sensors were obtained for class 3, and the lowest were found for class 1. Higher average readings for individual sensors were obtained for all classes in the wooden chamber (above 0.8 V for TGS826, TGS 2602, and TGS2603 for class 3), and lower results were found in the Styrofoam chamber (below 0.7 V for TGS826, TGS 2602, and TGS2603 for class 3) (Figure 6 and Figure 7).

The Monte Carlo cross-validation 25 test for class vs. all variants showed that it is possible to achieve separability in most classes at a satisfactory level of more than 60% using various classifiers. In addition, it was noted that the application of baseline correction allowed improving upon this result. The weakest separation was in class 17, where acc_balanced was usually below 0.6, and a quite low acc_class value of less than 0.3 was obtained. Class 4 obtained an accuracy balanced in Variant 1 at a borderline of 0.6, but after applying baseline correction, the value improved to almost 0.7 (Table 3 and Table 4).

Based on the analyses performed, nb was identified as the best classifier for the class vs. all comparison, which was a preview the results obtained for this classifier for each class and variant. The best distinguishing results in both the wooden chamber and Styrofoam chamber by the device were for the empty chamber and comb with both types of bee brood. Here, with baseline correction, acc balances of 0.7 or more were obtained. The class of an empty comb did not separate as well but was still at a satisfactory level (acc balanced above 0.6). Poorly recognized by the device were the combs with food (acc balanced below 0.57) (Table 5 and Table 6).

The Monte Carlo cross validation 25 class vs. class test with Variant 2 gave us surprising results. It turned out that the MCA-8 device was able to distinguish between an empty comb and a comb with brood with an accuracy of more than 83%, but the type of brood was poorly recognized by the device (acc_balanced 0.51 in the wooden chamber and 0.58 in the Styrofoam chamber). Satisfactory results were also obtained in distinguishing between an empty comb and a comb with food. In the Styrofoam chamber, the efficiency ranged from 62% (empty comb vs. comb with sugar syrup) to 72% (empty comb vs. comb with bee bread). In the wooden chamber, the empty comb and the comb with syrup were distinguished at a rate of 72%. Only the recognition efficiency of the comb with bee bread was low (acc_balanced 0.57). The differentiation of food types between each other was as poor as that of the brood types (acc balanced 0.49 in the wooden chamber and 0.57 in the polystyrene chamber) (Table 7).

## 5. Discussion

Our research introduces concepts that are entirely original and unprecedented in their character, offering a fresh perspective in the field. As for the usage of semiconductor gas sensors in beekeeping, thus far, the possibility of detecting dangerous bee and brood diseases with them has been analyzed [26,27,28,40], as well as the effectiveness in recognizing the status of worker bees [25] or honey varieties [41]. For the first time, it has been investigated whether the gas sensor matrix is able to recognize the contents of honey bee comb cells. By analyzing the already raw results of the sensor readings, we found that the classes were separated quite well. We were surprised that the readings of most sensors were higher for the brood comb than for the food comb. In an organoleptic test, carbohydrate food in the form of congealed sugar syrup is indeed not quite aromatic, but the comb with bee bread in the perception of the human nose gives a pungent and sour smell. It was not excluded that the higher values for the sensor readings were due to the fact that cells with brood are covered with a highly aromatic layer of propolis [15]. We observed that often the highest readings were obtained for the TGS826, TGS 2602, and TGS2603 sensors. In our other tests, the sensors from theTGS2xxx series in particular were also more sensitive [26]. These are newer-generation sensors targeting more complex gas mixtures.

We have also shown that it is sealed brood that elicits a higher sensor response than open brood. We expected the opposite result here. We were convinced that the volatile component profile of open brood, due to its components and lack of wax cappings, would activate the sensors more effectively. Perhaps we underestimated the power of pheromones not detectable with the human nose [5]. A sealed brood intensely produces pheromones, as this is a way to communicate with worker bees [42].

Observations of the raw data readings clearly turned into the results of the Monte Carlo cross-validation 25 analysis. In this, 14 methods of classifying the obtained data were compared. The naive Bayes classifier (nb) proved to be the most effective method. The use of differential baseline correction (Variant 2) further strengthened this efficiency [43,44]. Here, we showed that the MCA-8 device with a matrix of six TGS sensors distinguished an empty chamber and a comb with sealed and open brood from other classes at a rate of more than 70%. It distinguished empty combs from other classes with an accuracy above 62%. The comb with food proved to be the most difficult one to detect, with the accuracy balanced below 0.6 in both comb chambers.

Observation of the results for the Monte Carlo cross-validation 25 test with a combination of a class vs. another class showed us that in most cases, the MCA-8 device could easily distinguish between an empty comb and a comb with contents. Significantly more sensitive were the sensors in relation to the combs with brood. In both the wooden chamber and the polystyrene chamber, this classification accuracy was above 83%. On the other hand, we obtained low results for distinguishing between a comb with sealed brood and a comb with open brood. Therefore, for the present moment, we see that for the device, these classes are indistinguishable. In practice, this is not a problem because the most important thing for the beekeeper is whether brood has appeared in the bee nest, and with the MCA-8 device, the beekeeper could monitor this.

When it came to distinguishing between empty combs versus combs with food, in most cases, we obtained an accuracy above 62%. Only in the wooden chamber when comparing classes 15 and 17 was the balanced accuracy at 0.57. Also low was the differentiation of combs with carbohydrate food from combs with bee bread. In practice, the beekeeper will expect to know if honey has appeared in the comb. We analyzed the efficiency of detecting in the comb cells the presence of bee bread and carbohydrate food in the form of a little aromatic sugar syrup. Bee bread is formed from pollen. Pollen from different plants exhibits an odor specific to the plant species [45,46]. At the same time, fresh pollen smells noticeably more intense. During the drying process, some of the volatile compounds volatilize under the influence of elevated temperatures, causing the dried pollen to exhibit a less intense aroma. During the storage of pollen in the comb cells and pouring honey over them to create bee bread, it is likely that the pollen is weathered and loses its aroma. In fact, it should be remembered that the entire process of making bee bread occurs at the temperature of the bee nest, which is 35 °C. Thus, semiconductor gas sensors were entitled to react poorly to combs with bee bread and sugar syrup. Based on the results, we see potential in using semiconductor gas sensors to detect food in bee combs, particularly if they will have honey, a product of botanical origin which is richer in volatile gas fractions [3,4]. The next step in our research should therefore be to see how the MCA-8 device will respond to the different varieties of honey collected in combs.

## 6. Conclusions

Performing a baseline correction yielded a clear separation of classes, and we found that the highest sensor readings were obtained for the comb with brood, while the lowest results were for an empty chamber. We showed that the MCA-8 device in the Styrofoam chamber recognized classes quite well, with the accuracy ranging from 71 to 82%. In the wooden chamber, the results were unsatisfactory for the comb with carbohydrate food and comb with bee bread, where the accuracy fell below 60%. The device successfully showed that the comb was not empty. It was particularly effective in distinguishing an empty comb from a comb with brood, achieving an accuracy of over 83%. Lower classification accuracy was observed when distinguishing an empty comb from a comb with food. We did not obtain satisfactory results when distinguishing between the types of brood or types of food present in the comb cells, with the accuracy falling below 60% and the device being least reactive to the comb with bee bread. The naive Bayes classifier (NB) proved to be the most effective classifier for the research problem presented.

The MCA-8 device reliably identified non-empty comb cells, although distinguishing between the various contents, particularly sealed brood versus open brood and carbohydrate food versus bee bread, remains challenging. Despite these limitations, the outcomes are sufficiently encouraging to anticipate that with additional data, the detector’s capability to discriminate between the comb’s contents will improve. Our research has exploratory character. The preliminary results are so satisfactory that we see potential in the MCA-8 device for practical use in beekeeping as a tool to inform the beekeeper about the situation in a bee colony. However, in order to achieve this goal, it is necessary to conduct further research, especially under variable field conditions, and train classifiers.

## Figures and Tables

**Table 1:** The characteristics of the semiconductor gas sensors which were used in the multi-sensor matrix [26] (https://www.figaro.co.jp/en (2023)).

Sensor	Substances Detected
TGS823	Organic solvent vapors
TGS 826	Ammonia
TGS 832	Chlorofluorocarbons
TGS 2600	Gaseous air contaminants
TGS 2602	VOCs and odorous gases
TGS 2603	Amine-series and sulfurous odor gases

**Table 2:** The number of samples tested in each class and number of correct measurements.

Class	Kind of Sample	Samples Tested	Correct Measurements
1	Empty chamber	15	13
3	Comb with sealed brood	10	9
4	Comb with open brood	10	9
15	Empty comb	10	8
16	Comb with carbohydrate bee food	10	10
17	Comb with bee bread	10	7
Total		65	56

**Table 3:** The results of the Monte Carlo cross-validation 25 test for the wooden chamber, with an indication of the classifier for which the highest $b_{acc}$ value was obtained.

	Variant 1	$b_{\mathrm{acc}}$	${\mathrm{acc}}_{\mathrm{all}}$	${\mathrm{acc}}_{\mathrm{class}}$	Variant 2	$b_{\mathrm{acc}}$	${\mathrm{acc}}_{\mathrm{all}}$	${\mathrm{acc}}_{\mathrm{class}}$
**1 vs. all**	nb	0.798	0.846	0.750	lg	0.796	0.892	0.700
**3 vs. all**	3nn	0.746	0.906	0.587	nb	0.832	0.837	0.827
**4 vs. all**	3nn	0.589	0.937	0.240	nb	0.692	0.757	0.627
**15 vs. all**	1nn	0.777	0.894	0.660	2nn	0.653	0.906	0.400
**16 vs. all**	nb	0.644	0.874	0.413	1nn	0.686	0.851	0.520
**17 vs. all**	rf	0.521	0.981	0.060	1nn	0.546	0.851	0.240

**Table 4:** The results of the Monte Carlo cross-validation 25 test for the Styrofoam chamber, with an indication of the classifier for which the highest $b_{acc}$ value was obtained.

	Variant 1	$b_{\mathrm{acc}}$	${\mathrm{acc}}_{\mathrm{all}}$	${\mathrm{acc}}_{\mathrm{class}}$	Variant 2	$b_{\mathrm{acc}}$	${\mathrm{acc}}_{\mathrm{all}}$	${\mathrm{acc}}_{\mathrm{class}}$
**1 vs. all**	nb	0.733	0.855	0.610	nb	0.755	0.720	0.790
**3 vs. all**	nb	0.781	0.923	0.640	nb	0.819	0.851	0.787
**4 vs. all**	2nn	0.602	0.857	0.347	nb	0.713	0.866	0.560
**15 vs. all**	3nn	0.749	0.917	0.580	3nn	0.654	0.849	0.460
**16 vs. all**	1nn	0.689	0.871	0.507	lg	0.739	0.837	0.640
**17 vs. all**	1nn	0.583	0.907	0.260	1nn	0.604	0.928	0.280

**Table 5:** Results of Monte Carlo cross-validation 25 test for wooden chamber for naive Bayes classifier method (nb).

	Variant 1	$b_{\mathrm{acc}}$	${\mathrm{acc}}_{\mathrm{all}}$	${\mathrm{acc}}_{\mathrm{class}}$	Variant 2	$b_{\mathrm{acc}}$	${\mathrm{acc}}_{\mathrm{all}}$	${\mathrm{acc}}_{\mathrm{class}}$
**1 vs. all**	nb	0.798	0.846	0.750	nb	0.697	0.625	0.770
**3 vs. all**	nb	0.770	0.820	0.720	nb	0.832	0.837	0.827
**4 vs. all**	nb	0.609	0.817	0.400	nb	0.692	0.757	0.627
**15 vs. all**	nb	0.750	0.920	0.580	nb	0.621	0.763	0.480
**16 vs. all**	nb	0.644	0.874	0.413	nb	0.573	0.720	0.427
**17 vs. all**	nb	0.482	0.904	0.060	nb	0.429	0.757	0.100

**Table 6:** Results of Monte Carlo cross-validation 25 test for Styrofoam chamber for naive Bayes classifier method (nb).

	Variant 1	$b_{\mathrm{acc}}$	${\mathrm{acc}}_{\mathrm{all}}$	${\mathrm{acc}}_{\mathrm{class}}$	Variant 2	$b_{\mathrm{acc}}$	${\mathrm{acc}}_{\mathrm{all}}$	${\mathrm{acc}}_{\mathrm{class}}$
**1 vs. all**	nb	0.733	0.855	0.610	nb	0.755	0.720	0.790
**3 vs. all**	nb	0.781	0.923	0.640	nb	0.819	0.851	0.787
**4 vs. all**	nb	0.503	0.860	0.147	nb	0.713	0.866	0.560
**15 vs. all**	nb	0.604	0.829	0.380	nb	0.643	0.726	0.560
**16 vs. all**	nb	0.554	0.894	0.213	nb	0.570	0.846	0.293
**17 vs. all**	nb	0.502	0.864	0.140	nb	0.480	0.880	0.080

**Table 7:** The results of Monte Carlo cross-validation 25 test for wooden and polystyrene chambers for svm_ sigmoid method for Variant 2 with class vs. other class.

Class I	Wooden	*b*	acc	acc	Styrofoam	*b*	acc	acc
vs. Class II	Chamber	${}_{\mathrm{acc}}$	${}_{\mathrm{class}I}$	${}_{\mathrm{class}\mathrm{II}}$	**Chamber**	${}_{\mathrm{acc}}$	${}_{\mathrm{class}I}$	${}_{\mathrm{class}\mathrm{II}}$
**3 vs. 15**	svm_ sigmoid	0.880	0.760	1.000	svm_ sigmoid	0.938	0.890	0.987
**4 vs. 15**	svm_ sigmoid	0.890	0.780	1.000	svm_ sigmoid	0.832	0.690	0.973
**3 vs. 4**	svm_ sigmoid	0.505	0.530	0.480	svm_ sigmoid	0.575	0.610	0.540
**15 vs. 16**	svm_ sigmoid	0.723	0.947	0.510	svm_ sigmoid	0.620	0.640	0.600
**15 vs. 17**	svm_ sigmoid	0.573	0.920	0.227	svm_ sigmoid	0.727	0.947	0.507
**16 vs. 17**	svm_ sigmoid	0.493	0.840	0.147	svm_ sigmoid	0.570	0.940	0.200
